# The Bio-Engineering Approach for Plant Investigations and Growing Robots. A Mini-Review

**DOI:** 10.3389/frobt.2020.573014

**Published:** 2020-09-24

**Authors:** Barbara Mazzolai, Francesca Tramacere, Isabella Fiorello, Laura Margheri

**Affiliations:** ^1^Center for Micro-BioRobotics, Istituto Italiano di Tecnologia, Pontedera, Italy; ^2^The BioRobotics Institute, Scuola Superiore Sant'Anna, Pisa, Italy

**Keywords:** bioinspired robotics, soft robotics, growing robots, plants biology, smart materials, bioengineering

## Abstract

It has been 10 years since the publication of the first article looking at plants as a biomechatronic system and as model for robotics. Now, roboticists have started to look at plants differently and consider them as a model in the field of bioinspired robotics. Despite plants have been seen traditionally as passive entities, in reality they are able to grow, move, sense, and communicate. These features make plants an exceptional example of morphological computation - with probably the highest level of adaptability among all living beings. They are a unique model to design robots that can act in- and adapt to- unstructured, extreme, and dynamically changing environments exposed to sudden or long-term events. Although plant-inspired robotics is still a relatively new field, it has triggered the concept of growing robotics: an emerging area in which systems are designed to create their own body, adapt their morphology, and explore different environments. There is a reciprocal interest between biology and robotics: plants represent an excellent source of inspiration for achieving new robotic abilities, and engineering tools can be used to reveal new biological information. This way, a bidirectional biology-robotics strategy provides mutual benefits for both disciplines. This mini-review offers a brief overview of the fundamental aspects related to a *bioengineering approach in plant-inspired robotics*. It analyses the works in which both biological and engineering aspects have been investigated, and highlights the key elements of plants that have been milestones in the pioneering field of growing robots.

## 1. Introduction

How we see plants has changed significantly, as has the importance of protecting them for the benefit of the entire terrestrial ecosystem (Baluška and Mancuso, 2020). From an ecological role and evolutionary path, plants are producers in the food net of an ecosystem. They are photoautotroph organisms, so able to self-produce organic compounds by using mineral substances through photosynthesis. By exploiting substances directly from air and soil, they “do not need traditional locomotion,” but they evolved a number of singular strategies to interact with the environment, including complex movements, sensing, growing and propagation. New technologies, such as time-lapse recording, have demonstrated such abilities both above and below ground (Vincent et al., 2011; Silverberg et al., 2012; Vlad et al., 2014; Poppinga et al., 2015; Guerra et al., 2019; Rambaud-Lavigne and Hay, 2020). Also robotics has contributed to this change in perspective by starting to mimic plants at both components and system level (Mazzolai et al., 2010, 2011; Sadeghi et al., 2014, 2020; Hawkes et al., 2017; Nahar et al., 2017; Wooten and Walker, 2018; Must et al., 2019; Bolt et al., 2020; Geer et al., 2020).

This mini-review focuses on the vision of “science for robotics and robotics for science,” to highlight the results of a bioengineering approach in simultaneously driving innovative technological design and obtaining new biological insights.

## 2. From Plants to Robots

Plants are sessile organisms, and this means that they spend their entire lives at the site of seed germination. They have thus evolved a high level of plasticity enabling them to thrive, adapt and respond to changing conditions and survive under stress (Karban, 2008). Due to their exceptional adaptability, plants are the first living beings to colonize hostile environments, and have the unique capability to live contemporary in two different environments (e.g., soil and air, or water and air; Niklas and Spatz, 2012). These behaviors are linked to a complex and dynamic interaction between their morphology, distributed sensory-motor control, and the environment, which in turn represent the basic principles of what is called “morphological computation”(Laschi and Mazzolai, 2016): a modern perspective on intelligence in which the physical body has a primary role (Paul, 2006; Pfeifer and Bongard, 2007) and the behavior depends strongly on the mechanical properties, the form/morphology, and the arrangement of the perceptual, motor and “processing units” (Zambrano et al., 2014).

Plants are thus the perfect candidates to be a model to deal with a key challenge in robotics: the capacity to function in unstructured environments. This skill requires heightened abilities of perception, efficient use of energy resources, and high adaptability to dynamic environments and changing situations. Plants offer several ideas for designing innovative technologies, such as: (1) indeterminate growing capabilities; (2) movements without muscles; (3) structural materials with morphological adaptability and variable stiffness; (4) distributed intelligence and sensory systems; (5) anchoring/attachment strategies; (6) intra-system and inter-system communication; and (7) energy-saving mechanisms. Belowground, plants represent the best example among living beings for efficient soil non-destructive and capillary exploration. They have a network of growing and branching roots, whose tips are highly sensorized and efficiently move the soil volume and search for nutrients. Aboveground, plants are a unique model for the design of low-mass low-volume robots capable of anchoring themselves, negotiating voids, and climb where current climbing robots based on wheels, legs, or rails would get stuck or fall.

Table 1 reports the biological features, measurements and characterization methods, biological specifications, and plant-inspired robotic solutions discussed throughout the following sections.

**Table 1:**
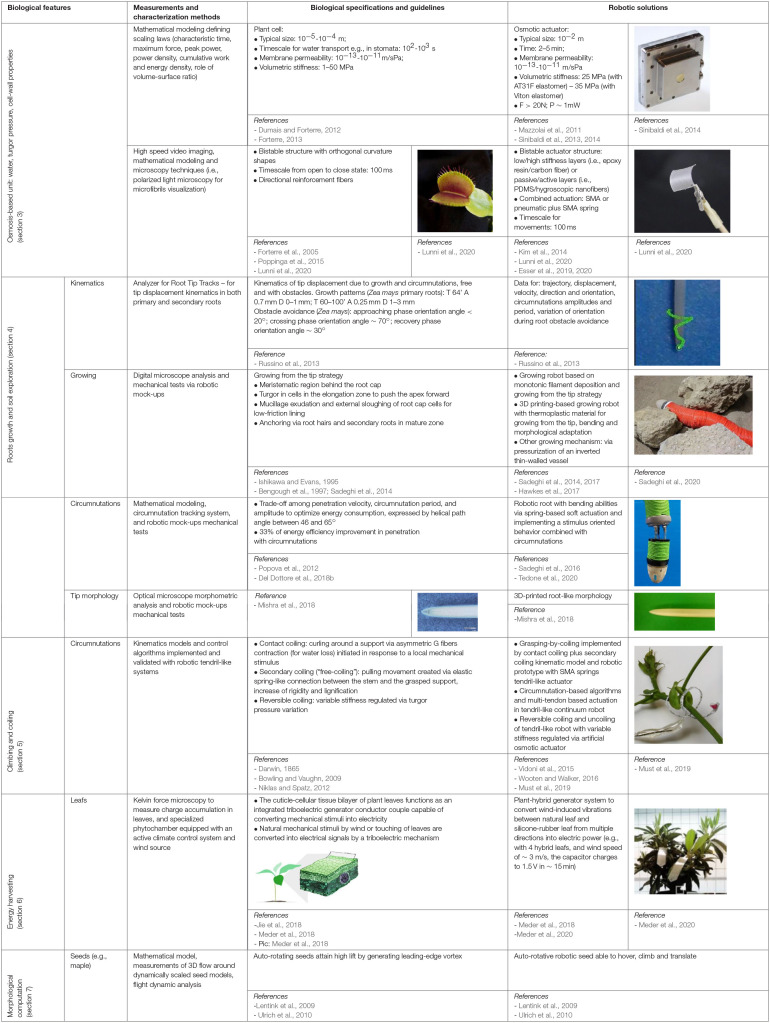
Summary of plants' biological features, measurements and characterization methods, biological specifications, plant-inspired robotic solutions.

## 3. Movements in Plants Without Muscles

From the growing of shoots and roots, to the opening and closing of stomata at the leaf surface, to the rapid snapping of carnivorous plants and the explosive launch of seed pods, plants have evolved a remarkable range of mechanisms to generate motions without the need for a muscular structure (Darwin, 1880; Gilroy and Masson, 2008; Forterre, 2013; Jung et al., 2014; Geitmann, 2016; Echevin et al., 2019; Morris and Blyth, 2019).

### 3.1. Water Transport

Plants turn out in a perfect hydraulic engine. At the macro level, plants exploit a sophisticated strategy, relying on water potential gradient, for moving water from the soil (i.e., at soil level ≈ −0.3 MPa) to the leaves (i.e., at the leaf level ≈ −7.0 MPa; McElrone et al., 2013). At the cell level, plants draw the water in and out of their cells using the osmotic gradient across semipermeable membranes. The turgor pressure thus changes creating a local change in the cellular volume and tissue stiffness, and by exploiting the thin and stiff cell-wall features, enables the large-scale tissue deformations required for motion (Dumais and Forterre, 2012). The characteristics of the water flow induced by gradients of water potential, the level of turgor pressure, and the mechanical properties of cell-wall deformation, are the key elements of water-driven movements in plants.

In roots, the movement of primary growth is characterized by the expansion of the cells facilitated by water uptake generating turgor pressure to inflate the cell and stretch the walls (Taiz and Zeiger, 2002). Plants are thus able to penetrate and explore soil in a non-destructive way. Such mechanism was first investigated from a robotics point of view in Mazzolai et al. (2011), with an osmotic actuation module implementing electro-osmosis by three cells separated by pairs of semipermeable osmotic membranes and ion-selective membranes, individually coupled with a piston mechanism. To aid the robotic design, Sinibaldi et al. (2013) followed a bioengineering approach to model the dynamics of osmotic actuation and represent a formal expression of scaling laws for the physical parameters necessary for the actuation strategy: characteristic time, maximum force, peak power, power density, cumulative work and energy density, role of volume-surface aspect ratio. This model was then exploited to design a forward osmosis-based actuator (Sinibaldi et al., 2014), fabricated on the basis of an analysis of plant movements, plant cell characteristics, and osmotic actuation modeling. The system has a typical size of 10 mm, produces forces above 20 N, with a power consumption in the order of 1 mW, and a characteristic time of 2–5 min.

### 3.2. Elastic Energy

To obtain faster movements, plants exploit instability and fluid-solid coupling together with hydraulic mechanisms: elastic energy is first stored in the cell walls by means of a water flow, and released suddenly when a critical threshold of energy barrier is surpassed (Forterre et al., 2005; Dumais and Forterre, 2012). The rapid movement of leaf closure in the carnivorous plant *Dionaea muscipula* (Venus flytrap) derives from the accumulation of elastic energy in the leaves, driven by swelling and shrinkage coupled with a double curvature geometry of lobes. This allows a snap-buckling mechanism of 100 ms after the initial trigger stimulus. These features inspired a flytrap-like robot described in Kim et al. (2014) which can reach rapid speed motion (~100 ms) and large deformations (18 m^−1^). The system consists of an asymmetrically laminated carbon fiber prepreg (CFRP), which acts as a bistable artificial leaf, and a shape memory alloy (SMA), which acts as triggering actuator to induce the snap motion. Differently, Esser et al. (2019) combined different actuation systems (pneumatic, plus SMA spring) to translate the principles of movements of Venus flytrap and waterwheel plant in systems able to response to different environmental triggers (heat, moisture or magnetic stimuli). More recently, polarized light microscopy revealed the presence of microfibrils reinforcing the leaf, running perpendicular to its midrib in the upper and lower epidermis. This additional feature, integrated with bistability, inspired the design of an artificial hygroscopic bistable system, obtained by bonding prestretched poly(dimethylsiloxane) – PDMS layers prior to depositing electrospun polyethylene oxide (PEO) nanofibers (Lunni et al., 2020). The Venus flytrap is an interesting model for robotic components and artificial materials, which have been recently deeply reviewed by Esser et al. (2020).

## 4. Below-Ground Mobility: Strategies and Morphological Features of Roots for Soil Exploration

### 4.1. Moving-By-Growing

The movement of roots inside the soil occurs by adding new cells to the apex. This strategy allows minimizing the resistance forces during penetration, and is helped by lateral hairs and diameter expansions that keep the whole system anchored. The roots' strategy of *growing from the tip* is a key specification for the development of robotic systems for soil exploration. To quantify the influence of this mechanism during penetration, Tonazzini et al. (2013) used a physical robotic demonstrator and showed that the growth from the tip reduces penetration energy from 20 to up 50%, depending on the initial depth. Following these quantitative analyses, Sadeghi et al. (2014) designed the first root-like system implementing a growing mechanism by means of a monotonic process that continuously adds new material to the base of the tip, in the form of a layer, and pushes forward the tip itself layer-by-layer.

This concept triggered the idea of *moving-by-growing*, paving the way for growing robots: by integrating a miniaturized 3D printer into the tip of a root-like device and using a thermoplastic filament, the body of the root-like system can be created layer-by-layer thus replicating the natural mechanism of cell deposition and consolidation that occurs in plants growth (Sadeghi et al., 2017). The concept of growing system has been approached also using other technologies. For example, the robot described in Hawkes et al. (2017) grows via pressurization of an inverted thin-walled vessel.

### 4.2. Circumnutations

To further enhance exploration abilities, circular movements (known as “circumnutations”) are performed by the root tip due to a combination of internal factors and external factors (i.e., gravitropism, Brown, 1993; Stolarz, 2009; Migliaccio et al., 2013). Circumnutations are a class of movements that are found in all plants organs, but in particular in those that are involved in growth (roots, shoots, branches, flower stalks) and generate elliptical or circular trajectories (Mugnai et al., 2015).

To quantify the characteristics of root circumnutations in soil for robotic design purposes, Popova et al. (2012); Del Dottore et al. (2018b), and Tedone et al. (2020) have proposed a methodology for the analysis of the movement, including a time-lapse videos observation (in air and in soil) and the study of tip kinematics using the “Analyser for Root Tip Tracks” (ARTT, Russino et al., 2013), which combines a segmentation algorithm with additional software imaging filters in order to realize a 2D tip detection. Measurements of the growing speed and circumnutation amplitude were extracted to implement the circumnutation behavior in a soft robotic root that bends using soft spring-based actuators (Sadeghi et al., 2016). Experiments in the air with the robotic root were performed to demonstrate that the system can follow an external stimulus while performing a circumnutation movement, similarly to a natural root. Additional experiments have been performed in a soil-like testbed showing that the use of plant root-like circumnutations improves the efficiency of penetration (33%) compared to moving directly forwards with no circular movement. In line with these results, additional investigations using “robophysical” modeling have revealed the benefits of tip nutation movements for navigating obstacles and exploring heterogenous terrains (McCaskey et al., 2019; Taylor et al., 2020).

### 4.3. Roots Morphology

To better understand the role of morphology of roots in soil, high-resolution imaging methods, such as micro-CT (Kaestner et al., 2006; Tracy et al., 2010) can be used to investigate natural roots and thus obtain a 3D reconstruction for bioinspired design insights. Mishra et al. (2018) used imaging capture of *Zea mays* roots via an optical microscope. The aim was to extract the morphological features of the tip profile and implement a 3D CAD model to guide the design and fabrication of 3D printed root-like probes. These devices, with different diameters and shapes, were compared in terms of energy consumption and penetration force via experimental tests in real soil and discrete element simulations, demonstrating the higher penetration performance of the bioinspired tip profile with respect to the other ones.

## 5. Above-Ground Movements: Remarkable Abilities in Climbing Plants

Climbing plants show interesting abilities to grow search for a support, and then attach, anchor or coil themselves onto it. They use several types of movements, including circumnutations, and exploit their tactile perception, adhesive properties, and ability to change their mechanical and morphological properties (Rowe and Speck, 2005, 2015). From a biomimetic perspective, recent reviews have focused on the attachment mechanisms (Burris et al., 2018) or those features that ensure highly flexible, soft and continuum robotic appendages (Fiorello et al., 2020). Tendrils and vines are particularly interesting in this framework, and specifically for the development of “searcher-like” robots (Wooten and Walker, 2016, 2018; Visentin et al., 2020).

### 5.1. Searching

Early stem growth, where young shoots extend into spaces and search for support, are known as “searchers.” An outstanding example of a light-mass searcher can be found in the climbing catus *Selenicereus setaceus* (Soffiatti and Rowe, 2020). Searchers often have a light but stiff structure, and are capable to extend across voids and perform circumnutations to improve the probability of touching a support (Gallenmüller et al., 2004). Circumnutations offer a valuable solution for adaptation algorithms in motion planning (Wooten and Walker, 2016, 2018). By analyzing the behavior of vines, circumnutation-based algorithm improves the performance of a tendril-like continuum robots, enabling efficient environmental contact and helping to guide and stabilize the system. Such strategy could be used, for example, in space for positioning sensors or exploring the surrounding environment (Mehling et al., 2006; Tonapi et al., 2014; Wooten and Walker, 2015; Nahar et al., 2017).

### 5.2. Coiling

In climbing plants, contact coiling starts when a local mechanical stimulus occurs in a tendril, which then start to curl around the support and grip to it. This coiling is associated with the presence of gelatinous fibers (“G fibers,” Bowling and Vaughn, 2009). A differential decrease in the water content of a G fibers-bilayer ribbon generates an asymmetric contractile force, which drives the coiling (Gerbode et al., 2012). Then, a secondary coiling (“free-coiling”) pulls the plant closer to the support, creating an elastic spring-like anchorage resistant to external loads or wind. The loss of water during the free-coiling phase leads to an increase in structural rigidity, or lignification, which prevents it from uncoiling. Contact and secondary coiling were investigated from a kinematic viewpoint in Vidoni et al. (2015) to design and develop a tendril-like system able to grasp-by-coiling.

In some cases, the natural mechanism of coiling is reversible, so if the support is not suitable, the tendril uncoils. This feature was implemented in a small-scale system by Must et al. (2019). The actuation strategy derives from a plant's capacity to actively control osmolyte gradients, and is based on the electrosorption of ions on flexible electrodes (in porous carbon), driven at low input voltages (1.3 V). A 1 cm electroactive unit reversibly controls the concentration of ions (acting as osmolyte) obtained through the dissolution of an electrolyte. This tendril-like soft robot, coupled to the control unit, performs coiling and uncoiling with reversible stiffening and actuation.

## 6. Energy Harvesting From Plant Leaves

In addition to their photosynthetic apparatus, leaves can also work as an integrated triboelectric generator and convert mechanical stimuli into electrical signals (Jie et al., 2018; Meder et al., 2018). Meder et al. (2018) used Kelvin force microscopy to investigate how the charge is generated at the leaving plant leaf and reported that the electric signal due to cuticle triboelectrification can be generated by natural mechanical stimuli, such as wind or contact with other leaves. This discovery inspired the first living plant-hybrid system that can convert wind energy into electricity. Tested under outdoor conditions in a controlled environment, these plant-hybrid generators convert wind from multiple directions to directly power light-emitting diodes or a digital thermometer (Meder et al., 2020).

## 7. Morphological Computation: Plant Seeds

Seeds are one of the most significant examples of morphological computation in the natural world. They provide a rich library of morphological and mechanical features optimized for passive take-off, flying, landing, and drilling (Fratzl and Barth, 2009). Although seeds lack active metabolism, hence no internal energy is produced, they are highly responsive to environmental conditions (e.g., temperature and humidity) and have anisotropic and reversible movements.

Environmental responsiveness is due to materials (Chambers and MacMahon, 1994; Burgert and Fratzl, 2009; Sadlo et al., 2018) and structural features (Abraham and Elbaum, 2013) of the seed tissues. As example, autorotating seeds of maples generate a surprisingly high lift by creating a stable leading-edge vortex as they descend (Lentink et al., 2009). Taking into account their geometries and flight trajectories, such capabilities can be used for the design of auto-rotative robotic samara air vehicles (Ulrich et al., 2010).

New fabrication technologies, like 4D printing, are allowing to advance multi-functional materials capabilities, such as in the case of biomimetic hygro-responsive composite polymer inspired by the reversible shape-changes of Bhutan pine (*Pinus wallichiana*) cone seed scales (Correa et al., 2020).

## 8. Conclusions

Plants as a model has been officially accepted within the robotics community, inspiring also new sensors (Sareh et al., 2014; Lucarotti et al., 2015; Su et al., 2015; Blandin et al., 2017; Cheng et al., 2018) and swarm communication strategies (Ciszak et al., 2012; Del Dottore et al., 2018a).

Besides, plant-inspired robots (Sadeghi et al., 2014), robophysical models (McCaskey et al., 2019), and behavior models (Agostinelli et al., 2020), have been crucial to validate hypothesis on plants' mechanisms, closing the circle of bio-robotics.

There is a new vision for bioinspired robots, in which robots are seen as environmentally responsible machines that can grow, adapt, and are built with recyclable, or biodegradable, or biohybrid materials (Yang et al., 2018; Mazzolai and Laschi, 2020).

To achieve this, we need to look at a more global level, investigating the strategies and synergies of natural organisms (Aartsma et al., 2017; Paudel Timilsena et al., 2020), and at how they are integrated harmoniously within the natural ecosystem. All these aspects need to be further studied with a multi-disciplinary approach to develop a new wave of environmentally-responsible robots.

Plants are not only a source of inspiration for current and future technological progress in an environmentally responsible integrated vision. Equally importantly, they are key to our future welfare. In order for us to better understand and protect the biodiversity of species and the whole global environment, it is imperative that we learn more about their features, as well as how they deal with the natural ecosystem.

## Author Contributions

BM and LM conceived the focus and the format of the mini-review. BM, LM, FT, and IF curated the existing bibliography, contributed to the table contents, and to the writing of the manuscript. All authors contributed to the article and approved the submitted version.

## Conflict of Interest

The authors declare that the research was conducted in the absence of any commercial or financial relationships that could be construed as a potential conflict of interest.
